# Accessing quality motorcycle helmets in Ghana: a qualitative inquiry

**DOI:** 10.1016/j.afjem.2026.101020

**Published:** 2026-10-24

**Authors:** Daniel Gyaase, Theresah Ivy Gyaase, Margie Peden, Pratishtha Singh, Jagnoor Jagnoor

**Affiliations:** a Injury Division, The George Institute for Global Health, Faculty of Medicine and Health, University of New South Wales, Sydney, Australia; b Department of Health Promotion and Disability Studies, Kwame Nkrumah University of Science and Technology, Kumasi, Ghana; c Global Injury Program, The George Institute for Global Health, Imperial College London, United Kingdom

**Keywords:** Helmet, Quality, Barriers, Facilitators

## Abstract

**Background:**

While helmet use among motorcyclists in Africa remains low, an equally critical issue is the quality of helmets in use. In many African countries, such as Ghana, poor-quality helmets are widely available in local markets, mainly due to a lack of helmet standards, weak import regulations, and poor enforcement. This study, therefore, explores the determinants and enablers of access to quality motorcycle helmets in Ghana.

**Methods:**

We conducted a qualitative inquiry between January and April 2024. We purposively sampled participants across three interest groups: motorcyclists, helmet vendors/retailers, and road safety practitioners. We collected data through in-depth, face-to-face interviews using semi-structured interview guides. We analysed the data using inductive thematic analysis.

**Results:**

We interviewed 41 participants: 16 motorcyclists, 19 helmet vendors/retailers, and 6 road safety practitioners. The analysis highlighted four interconnected system gaps and potential enablers: limited knowledge and awareness of helmet quality, limited availability of quality helmets, economic constraints, and the absence of regulations and enforcement. Enablers include public education and awareness of quality helmets, improved market availability, price controls and incentives for motorcyclists, and stronger regulation and enforcement. Together, these factors limit access to quality helmets, and a systemic regulatory gap in standards and enforcement exacerbates the problem.

**Conclusion:**

Improving access to quality helmets requires a coordinated approach. The first step is to address the regulatory gap by developing helmet standards. Without this, it is difficult to ensure market availability, raise public awareness, and make helmets more affordable.

**African Relevance**

• The study addresses a major and growing African road safety problem, as motorcycle use is rising rapidly across the continent and motorcycle injuries and deaths are contributing substantially to the rising burden of road traffic fatalities.
• The findings are significant for many African countries that have helmet laws, yet weak or missing helmet quality standards, conformity assessments, and market surveillance enable the widespread circulation of substandard helmets.
• The study highlights that merely increasing helmet use is not enough in African contexts if the helmets sold locally do not offer sufficient protection.
• The study highlights barriers like awareness, availability, affordability, and enforcement from an African systems view, relevant to other low- and middle-income countries facing similar transportation and regulatory issues.

## Introduction

Road traffic fatalities are a major health issue in Africa, with a 17% rise over the past decade, unlike other WHO regions, where fatalities have declined [1]. One key factor for this increase is the significant rise in motorcycle fatalities in this same period. Like the overall African trend, motorcycle fatalities significantly increased in Ghana from 210 in 2010 to 915 in 2021 [2]. For the first and second consecutive times in 2020 and 2021, motorcycle fatalities exceeded pedestrian fatalities in Ghana [2,3].

Helmets are a crucial safety measure for motorcyclists, as they reduce the risk of head injuries and fatalities in crashes. Evidence shows helmet use reduces fatal motorcycle injuries by 28 to 64%, head injuries by 58 to 60%, brain injuries by 47 to 74%, face injuries by 14 to 63% and neck injuries by 14 to 48% [[4], [5], [6]]. However, poor-quality, substandard, or improper use, such as strapping, reduces this protective effect. Like Ghana, many African countries have laws mandating helmet use for all motorcyclists. However, helmet use in many African regions remains very low [[7], [8], [9], [10], [11]], attributed to weak law enforcement, discomfort from heat and poor ventilation, and cultural practices [12]. Despite laws requiring helmets for all riders in Ghana, the actual use remains low [8,11,13], leading to preventable motorcycle injuries and fatalities.

Beyond low helmet use, helmet quality is a critical issue in Ghana, and many African countries. Even when riders wear helmets, they may be poor quality [13] and fail to provide optimal protection. A key challenge is the absence of country-specific motorcycle helmet standards and import regulations, which have allowed a wide range of unregulated helmets to enter the Ghanaian market. While studies have examined barriers to helmet use [[14], [15], [16]], less attention has focused on the quality of helmets worn by the few who do. Gyaase and colleagues in Ghana have reported that many helmets used by motorcyclists are of poor quality [17]. Because there is no helmet standard, Ghanaian motorcyclists cannot rely on official criteria to guide their helmet choices, and no compliance checks occur at points of entry. Therefore, it is important to understand stakeholders’ perspectives on the issues and challenges of accessing quality motorcycle helmets. This study aims to explore challenges to accessing quality motorcycle helmets and identify potential enablers to address them.

## Methods

### Study design

We conducted a qualitative inquiry to explore the determinants of access to quality motorcycle helmets in Ghana. In addition to motorcyclists, we conducted a stakeholder analysis to identify key interest groups related to motorcycle safety, including helmet vendors/retailers and road safety practitioners. This approach helped us gain an in-depth understanding of the subject from various interest groups. The reporting of this study adheres to the Standards for Reporting Qualitative Research (SRQR) criteria [18], and the SRQR checklist is included as a supplementary document. In this study, we defined quality motorcycle helmets by structural features, including an energy‑absorbing liner, a retention system, an energy-absorbing layer, and a hard outer shell. We used this definition as an analytical reference point in the absence of national helmet standards, and it has previously been used to assess helmet quality in Ghana [17] and Vietnam [19]. We therefore examined participants’ perceptions of helmet quality against this benchmark to illustrate the practical challenges faced by riders, vendors, and regulators in an unstandardised market.

### Setting

We recruited motorcyclists and helmet vendors/retailers from the Tamale Metropolitan Area of Ghana. The Tamale metropolis is situated in the northern part of the country. The metropolis has a population of 374,744 (49.4% males and 50.6% females). The most common modes of intra-city transport are motorcycles and shared tricycles. Over 80% of Tamale residents own either a bicycle or a motorcycle. Most inhabitants in and around the Central Business District use motorcycles as their main mode of transport. In the Northern Region, where Tamale is located, motorcycle registrations account for approximately 90% of all registered vehicles [20]. We recruited road safety practitioners from national and regional levels.

### Participants

Participants included motorcyclists, helmet vendors/retailers, and road safety practitioners. We included motorcyclists aged 18 years and above, vendors/retailers aged 18 years and above who sell helmets and related products, and road safety practitioners with over 3 years of experience. We excluded motorcyclists, vendors/retailers, and road safety practitioners who did not meet the inclusion criteria. Participants were purposively selected.

We recruited motorcyclists from public places such as fuel stations, washing bays, and markets. When motorcyclists stopped, the team approached them, explained the research process, objectives, and benefits, and asked whether they would like to participate. We gave those who agreed to participate a recruitment form to review and sign. When they signed the forms, we interviewed them on the spot because we could not reliably reschedule interviews with riders recruited in public places. For vendors and retailers, we visited shops that sell helmets and recruited them for thestudy. During these visits, we verbally outlined the study's objectives and benefits, and provided a recruitment letter for their consideration. If they agreed to participate, we scheduled an interview date and provided a consent form for them to sign. We conducted on-the-spot interviews with those who opted for it. Road safety practitioners included representatives from the Road Safety Authority, the Motor Transport and Traffic Department of the Ghana Police Service, and road safety consultants. We sent letters to the national head offices for approval for their participation and to allow regional heads to also participate in the study. Once approved, we visited the respective offices with the approval letters to schedule interview dates and times. During these visits, we provided the recruitment letter and consent form.

We did not estimate the sample size before the study began, in accordance with best practice for qualitative studies; however, we set a minimum number of participants for each interest group. For motorcyclists and helmet vendors, the minimum participant quota was 16, allowing the eight research assistants to interview 4 participants (2 motorcyclists and 2 helmet vendors/retailers). For road safety practitioners, the minimum participant quota was 5, including the directors of the national and regional offices of the institutions/agencies.

### Data collection

D.G. and eight trained research assistants with at least a tertiary qualification collected the data. Research assistants were trained for three days on the purpose of the study, recruitment, consent and conducting interviews. D.G. interviewed road safety practitioners, while the research assistants interviewed motorcyclists and helmet vendors/retailers, speaking both English and the local dialects of the study area.

We conducted in-depth, face-to-face interviews using a semi-structured guide to collect data. D.G. developed the interview guides based on the study's objectives, and they were revised by J.J. Probes and follow-up questions were used when necessary. Data collection spanned three months (January–April 2024), during which the team took field notes and audio recordings. Interviews lasted between 20 - 63 min and were conducted in a quiet, safe place chosen by the participant.

### Data analysis

Trained research assistants transcribed the recorded interviews verbatim in the local dialect and then translated them into English. No interviews were repeated, and participants did not receive transcripts or interim analyses for member checking. However, to ensure the transcripts were accurate for motorcyclists and helmet vendors, we sent the transcripts and corresponding audio to an academic in the study area who understands the local dialect for verification, as the primary researchers do not.

We analysed the data using an inductive thematic analysis approach. First, DG reviewed the available interview notes alongside the transcripts, noting initial ideas and potential codes related to determinants (grouped into challenges and enablers). DG and TIG independently read all transcripts multiple times to become familiar with the interviews' content and participants' tone. DG and TIG then coded collaboratively. The transcripts were manually coded. Each researcher (DG and TIG) segmented participants' responses into meaning units and generated initial codes for both system gaps and enablers. After independently coding, DG and TIG compared, discussed, and reconciled their codes and resolved any discrepancies. Themes were developed from semantically similar codes within each determinant grouping. The themes were further reviewed and discussed with PS, and through discussion with DG, the final themes were identified.

Several procedures enhanced the credibility and trustworthiness of the analysis. Research assistants received training before data collection, and we audio-recorded and transcribed interviews. For interviews conducted in local languages, an academic familiar with the local dialect independently checked transcripts and corresponding audio recordings to verify transcription and translation accuracy. DG and TIG independently familiarised themselves with the transcripts and collaboratively developed and reconciled codes, with disagreements resolved through discussion. We then reviewed the developing themes with PS. We also compared perspectives across motorcyclists, helmet vendors/retailers, and road-safety practitioners. We did not conduct participant member checking, which we acknowledge as a limitation. Transferability is supported by the detailed description of the study setting, sampling and participant groups; however, motorcyclists and retailers were recruited from Tamale, which limits direct geographic transferability to other regions of Ghana.

### Ethical approval

The study was a part of a large motorcycle study and received ethical approval from the University of New South Wales (UNSW) Human Research Ethics Committee (HREC), with approval reference number iRECS0826 and the Kwame Nkrumah University of Science and Technology (KNUST) Committee on Human Research, Publication and Ethics (CHRPE), with reference CHRPE/AP/1066/23. All procedures followed institutional guidelines and adhered to the ethical standards outlined in the 1964 Helsinki Declaration and its subsequent amendments or equivalent ethical principles.

## Results

We interviewed 41 participants: 16 motorcyclists, 19 vendors and retailers, and 6 road safety practitioners. Participants ranged in age from 20 to 63 years (motorcyclists), 25 to 59 years (vendors/retailers), and 48 to 66 years (road safety practitioners). Participants varied in education level, motorcycle use, and professional experience. Motorcyclists included private riders and riders who used motorcycles for daily commuting or income-generating activities; vendors ranged from informal roadside sellers to more established shops; and road safety practitioners had regional or national responsibilities. These differences provided a broad view of how helmet quality is understood, supplied, purchased, and regulated in Ghana.

Our analysis identified four themes shaping access to quality motorcycle helmets in Ghana: (i) lack of awareness and knowledge around helmet quality, (ii) limited availability of certified helmets in local markets, (iii) economic constraints and affordability, and (iv) lack of regulations and enforcement. These themes were closely connected, reflecting how individual-level perceptions and choices were embedded within broader structural and market conditions. For instance, limited awareness and weak regulation perpetuated the circulation of low-quality helmets, while high costs and poor market availability further constrained riders’ ability to make safer choices (Table 1).

**Table 1:** System gaps and enablers in accessing quality helmets: views from motorcyclists, helmet retailers and road safety practitioners in Ghana.

**Views of the participants**		
**Categories**	**Subcategories**	**Codes**
**System gaps**	Lack of knowledge and awareness	Lack of knowledge and awareness of quality helmets
	Limited availability of quality helmets	Market composition (saturation with poor-quality helmets)
		Urban and rural access
	Economic constraints	High cost of quality helmets
		High taxes and duties on importation
	Lack of regulations and enforcement	Absence of a standard for helmets
		Weak enforcement
		Policy weakness
**Enabler**	Public education and awareness	Public education and awareness on quality helmets
	Improving market availability and distribution	Importing more quality helmets
		Quality helmets at vantage points
		Bundling quality helmets with motorcycle purchases
	Economic incentives and price controls	Price reduction
		Tax incentives for importers
		Price incentives
	Regulations and enforcement	Enhance import and market regulations
		Enforcement
		Mandatory certified helmets with motorcycle sales and registration

### Category of barriers

Participants categorised the reported challenges into four: knowledge and awareness, availability, affordability, and regulations. Each subcategory is explained below.

### Lack of knowledge and awareness of quality helmets

Across participant groups, limited knowledge of what constitutes a quality helmet emerged as a foundational challenge. Riders often relied on appearance, weight, price, or source as proxies for quality, while retailers and road safety practitioners noted that this lack of consumer knowledge reduced demand for safer helmets and allowed low-quality products to remain common. One motorcyclist noted:

“*Lack of information. For instance, if you bring three helmets and put them down, I wouldn’t be able to tell you which one is quality………. So, lack of information. We are not educated about the different types of helmets and their durability or the quality*” (Motorcyclist 3).

*“The biggest challenge from our motor riders not getting the correct helmet is ignorance. They don't know which one is quality... The motor companies or sellers are convincing them that this thing I’m selling is good… They give them the wrong education and sell them the wrong crash helmets”* (Road safety practitioner 6)

### Limited availability of quality helmets

Participants reported that quality helmets were difficult to find, particularly outside urban centres. The market was described as dominated by poor-quality, second-hand, or unverified products, while the few perceived quality helmets were harder to source and often more expensive. One retailer stated:

*“The availability is not there at all; sometimes you will struggle to get a helmet, so that is why some of [the helmets] are expensive”* (Retailer 2)

*“You see, helmets that come with a motorbike are always considered to be quality. So, when we misplace these quality helmets, replacing them becomes difficult, and then we resort into buying some of these roadside inferior helmets.”* (Motorcyclist 16)

Regional disparities compounded this problem, with participants noting that riders in smaller towns or rural areas had fewer options and often relied on whatever helmets were locally available.

*“When you are in Tamale here, and you are buying a helmet from Accra, you will not know if the helmet is standard or not standard, because of the distance”* (Retailer 8)

### Economic constraints

Affordability strongly influenced purchasing decisions. Motorcyclists, retailers, and road safety practitioners consistently stated that quality helmets were often priced beyond riders’ means, leading many to choose cheaper products despite recognising potential quality differences.

*“I think one of the challenges is cost. Cost in the sense that, you know, sometimes you go to the market, you want to buy a helmet, they tell you that, for example, this is GHȻ 200, and this is GHȻ 150, and this is GHȻ 70. As a rational consumer, I will go for the lowest.”* (Motorcyclist 7)

Retailers linked the high retail price of helmets to import duties, taxes, shipping, and distribution costs, which were passed on to consumers. This created a cycle in which quality helmets remained expensive and low-quality products continued to dominate the market.

*“…the duty is the cause of all the problems. You will pay about four to five times the cost of the goods you imported in duties at the port, how can you sell it at a cheap price? If you sell it at a cheap price, you will run at a loss and cannot import goods again”* (Retailer 17)

### Lack of regulations and enforcement

Participants identified the absence of national helmet standards as the central regulatory barrier. Without clear safety or certification criteria, importers and retailers could sell helmets of varying quality, and enforcement authorities lacked a practical basis for distinguishing standard from non-standard helmets. Participants noted:

*“There are no regulations on what should be imported and how they are sold. Therefore, anyone who brings their helmets sells them with no problem, whether it is good or bad, they sell.”* (Motorcyclist 12)

*“…And then, the police even the enforcement, what are they enforcing against? There is no standard. So that prescribed helmet you are talking about, I don’t know how the police will describe it and go and stand in court and prosecute someone for”* (Road safety practitioner 5)

Participants also described Ghana’s helmet market as largely unregulated, allowing substandard or second-hand helmets to circulate with minimal oversight. They further noted a policy gap in motorcycle sales and registration, where motorcycles may be sold without certified helmets, leaving riders to purchase cheaper alternatives.

*“Now, we have an open market where everybody is doing their business, because we don't have a streamlined system… everybody is bringing in what they want and what they think they can trade in.”* (Road safety practitioner 2)

### Category of enablers

Participants identified four main enablers: public education, improved availability and distribution, economic incentives, and stronger regulation and enforcement.

### Public education and awareness

Participants recommended public education that explains how to identify quality helmets and why certification matters. However, participants viewed education as most useful when linked to clear national standards, visible labelling, and coordinated messaging through road safety agencies, motorcycle dealers, retailers, and community outreach.

*“I think quality helmets can be made accessible by advertisement. So, if it’s been made known to the public through all the various platforms that this helmet is this and that, then riders can easily access it………... Or maybe, do some collaboration with the motor dealers where the quality helmets will be introduced to the motor dealers, where they can advertise them to their customers when they come to buy the motorbikes”* (Motorcyclist 3)

### Improving market availability and distribution

Participants emphasised that availability could improve if importers, dealers, and retailers supplied only certified helmets and made them easier to purchase at motorcycle dealerships, spare parts shops, and other common access points. They also recommended bundling certified helmets with motorcycle sales as a practical way to normalise access.

*“I think new motorbikes should come with two helmets. In the sense that one would serve as a spare. In case one is misplaced, you would still have another one to hold onto.”* (Motorcyclist 16)

To address affordability, participants recommended reducing import duties and taxes on certified helmets and introducing subsidies, vouchers, credit, or instalment payment options for riders. These measures were framed as ways to reduce the upfront cost of safer helmets without increasing the circulation of poor-quality products.

*“When the price reduces, it will be easy for us to access them.”* (Motorcyclist 1)

*“….implementing subsidies or voucher programs can help lower-income individuals purchase quality helmets at reduced prices” (Retailer 1)*

### Regulations and enforcement

Participants recommended a regulatory package that begins with national helmet standards and extends to import checks, market surveillance, dealer compliance, and enforcement. They also suggested requiring certified helmets at motorcycle sale, registration, or renewal to make access to quality helmets routine rather than optional.

*“There should be a law or standard that directs people to know the good quality helmet. This is what can make them more accessible in the market and for everyone”* (Retailer 12)

*“…Before you are assigned a motorcycle registration number, you have to show your mandated certified helmet….”* (Road safety practitioner 3).

## Discussion

This study explored the determinants of accessing quality motorcycle helmets in Ghana. Four interconnected themes were identified: knowledge and awareness about quality helmets, availability and market structure, economic factors, and regulations and enforcement. The findings suggest that the absence of national helmet standards sits at the centre of these challenges: it allows unregulated imports, weakens enforcement, limits consumer guidance, and contributes to a market dominated by poor-quality, counterfeit, or unverified helmets. The discussion therefore focuses on standard setting as the key policy entry point, while also considering education, affordability, distribution, and enforcement as complementary strategies.

Motorcyclists’ limited knowledge and understanding of quality helmets undermines their ability to make informed purchasing decisions. Participants consistently highlighted that most motorcyclists lack knowledge of what constitutes a quality helmet and base their purchasing decisions on factors such as price, appearance, size, or source. This pattern aligns with evidence from other LMICs: motorcyclists struggle to distinguish genuine from counterfeit helmets [21]. Prior to the study, Ghana had no national helmet standards; riders had no certified labels or criteria to guide their purchasing decisions. The results showed that some riders considered helmets supplied with motorcycles to be quality helmets, even though the market also includes second-hand and unverified products. This perception may reflect broader local assumptions that imported or previously used products from high-income countries have undergone quality checks and are therefore superior [22,23]. Without standards, these assumptions may shape both consumer demand and retailer/importer decisions, reinforcing a market in which second-hand or unverified helmets may be seen as safer despite limited evidence of their protective performance.

Participants stated the need for public education on helmet quality and how to identify it, to increase knowledge and improve awareness among riders. At the time of data collection (January–April 2024), however, Ghana did not yet have a national motorcycle-helmet standard to provide riders, retailers, importers, and enforcement agencies with a common technical criterion for helmet quality. Since data collection, the Ghana Standards Authority has issued GS 4512, Road Vehicles – Protective Helmets and Their Visors for Drivers/Riders and Passengers of Category L Vehicles. The policy implication of our findings has therefore evolved: rather than developing a standard as the next step, priority should now be given to effective implementation of the new standard, conformity assessment and market surveillance, enforcement at import and retail levels, and public communication that enables consumers to recognise compliant helmets. The new standard provides a concrete benchmark for consumer education and enforcement.

Quality helmets are limited in the local market, leading to widespread availability of poor-quality products, especially in rural and peri-urban areas. Riders in smaller towns or rural areas often have no choice but to buy whatever helmets are available, which are typically low-quality and offer minimal protection. Without standards and specifications, importers faced no restrictions and could import any products, with no clear guidance on what was allowed. In such an unregulated, open-market environment, the market becomes saturated with low-cost, low-quality imports, while high-quality helmets remain scarce. Since Ghana lacks local manufacturing capacity, importation is the only supply route, and importers face no obligation to ensure product quality. One practical way to improve availability is to ensure motorcycle dealerships and spare parts shops consistently stock only quality (standard) helmets. Improving availability will require policy shifts that will tie quality to distribution practices. This includes ensuring that motorcycle dealerships and spare parts retailers consistently stock certified helmets and bundling an approved helmet with every new motorcycle sale. Such measures would help normalise access to quality helmets at key points in the supply chain.

Economic barriers influence helmet purchasing decisions. Quality helmets are limited, and the few available ones, including some second-hand products perceived as quality (data not reported), are expensive. Although this study did not report it, most people who frequently use motorcycles, either for commercial purposes or daily commuting, have low socioeconomic status and cannot afford the quality helmets available. Helmets imported into Ghana from HICs (assumed to be high quality) face high Ghanaian customs duties, taxes, and shipping costs, which significantly raise retail prices. These costs are passed on to consumers (motorcyclists), raising retail prices and making such helmets less affordable for many motorcyclists. Our findings align with evidence that price is a crucial factor influencing helmet purchases in low-income settings. While a quality helmet may be cheap from the manufacturer, shipping, tariffs, and distribution costs raise the price, particularly in countries like Ghana, which lack local manufacturing capabilities [21]. In Ghana, a survey in the Northern region found that the median price of a standard helmet was roughly 1.5 days' average wage [24], making it difficult to afford. To bridge the gap, participants called for government intervention to lessen the financial burden on motorcyclists. These include reducing import duties and taxes, offering subsidies or voucher schemes for low-income riders, and considering quality helmets as tax-exempt essential goods.

Across themes, the absence of enforceable national helmet standards emerges as the foundational barrier, shaping knowledge, market supply, pricing, and enforcement. At the time of data collection in 2024, Ghana had no specific national motorcycle helmet standard against which helmets could be consistently assessed for safety performance and conformity. However, this finding should be interpreted in light of important regulatory developments since the study. In August 2026, the Ghana Standards Authority introduced GS 4512, which specifies requirements for protective helmets and visors for riders and passengers of Category L vehicles, including motorcycles. Ghana has since moved from standard development to implementation. These developments directly address one of the principal system gaps identified by participants and provide a regulatory basis for improving helmet quality in the Ghanaian market. Nevertheless, establishing a standard alone is unlikely to guarantee access to quality helmets. Effective implementation will require sustained import surveillance and conformity assessment, market surveillance to identify and remove non-compliant products already in circulation, adequate testing and certification capacity, and clear mechanisms that enable riders and retailers to identify compliant helmets. These measures should be accompanied by public education on the new standard and recognised certification or conformity markings, particularly because participants in this study reported limited ability to distinguish quality from poor-quality helmets. The introduction of the Road Traffic (Amendment) Act, 2025 (Act 1153) and the Road Traffic Regulations, 2026 (L.I. 2519), particularly the formalisation of commercial motorcycle operations, provides an additional opportunity to embed helmet-quality requirements within routine regulatory processes.

Although we recruited motorcyclists and helmet vendors/retailers in Tamale, some of the barriers identified have implications beyond the study setting because they relate to national regulatory and market systems. Helmet standards, product certification, import controls, and regulatory enforcement are determined at the national level, and road safety practitioners at regional and national levels also identified these issues. Nevertheless, the findings on riders’ experiences, affordability, availability, and the composition of the local helmet market should not be assumed to represent all parts of Ghana. Motorcycle use and helmet markets may differ between northern and southern Ghana. The study therefore supports national recommendations primarily for system-level regulatory issues, while further research across different regions is needed to determine whether the market and consumer-level barriers identified in Tamale are similarly experienced elsewhere.

Despite the significance of our study, it has limitations that readers should consider when interpreting the findings. First, we collected data primarily from motorcyclists and helmet retailers in the Tamale Metropolitan Area in northern Ghana, which may limit the geographic transferability of the results. However, Tamale represents a setting where motorcycles are the dominant mode of transport and where market conditions, regulatory oversight, and enforcement challenges closely resemble those in many peri‑urban and regional contexts across Ghana. Future research could extend this work by examining helmet markets and regulatory practices in other regions to further strengthen transferability. Also, despite efforts to ensure transcript accuracy, translating from local dialects into English may have reduced some depth.

Importantly, the challenges identified reflect systemic governance and market failures rather than individual shortcomings, reinforcing the need for national‑level regulatory action as a prerequisite for effective education, enforcement, and affordability strategies.

## Conclusion

This study shows that in Ghana, motorcyclists’ access to quality helmets is constrained by interacting deficits in knowledge, market availability, affordability, and, most fundamentally, the absence of enforceable helmet standards. Developing and enforcing national helmet standards is fundamental to addressing these gaps, and once in place, future research should evaluate implementation and test packages, such as public education, price measures, and enforcement strategies.

## CRediT authorship contribution statement

**Daniel Gyaase**: Conceptualisation, Investigation, Methodology, Project administration, Data curation, Formal analysis, Writing – original draft, reviewing and editing; **Theresah Ivy Gyaase**: Formal analysis, reviewing and editing; **Pratishtha Singh**: Formal analysis, reviewing and editing; **Jagnoor Jagnoor**: Conceptualisation, Methodology, Supervision, writing – original draft, writing – review and editing.

## Declaration of generative AI and AI-assisted technologies in the manuscript preparation process

In preparing this paper, the primary author used ChatGPT and Copilot to revise text and improve flow. After using these tools, the author reviewed and edited the content and takes full responsibility for the paper.

## Funding

DG is supported by the University International Postgraduate Award (UIPA) from the 10.13039/501100001773University of New South Wales (UNSW). JJ is supported by the 10.13039/501100000925National Health and Medical Research Council (NHMRC) as an emerging leader 1.

## Dissemination of results

The results of this qualitative inquiry will be presented during the stakeholders' consultation meeting on developing standards for helmets in Ghana. The abstract will also be submitted to conferences, particularly those related to road safety in Africa. Additionally, we will share the published paper with the public sector agencies responsible for road safety in Ghana, such as the National Road Safety Authority and the Ministry of Transport.

## Declaration of interests

The authors declare that they have no known competing financial interests or personal relationships that could have appeared to influence the work reported in this paper.
